# Organic Thermoelectric Multilayers with High Stretchiness

**DOI:** 10.3390/nano10010041

**Published:** 2019-12-23

**Authors:** Chungyeon Cho, Jihun Son

**Affiliations:** Department of Carbon Convergence Engineering, College of Engineering, Wonkwang University, Iksan 54538, Jeonbuk, Korea; sjhoon14@wku.ac.kr

**Keywords:** thermoelectric multilayers, layer-by-layer assembly, stretchable thin films, organic multilayers, power factor, carbon nanomaterials

## Abstract

A stretchable organic thermoelectric multilayer is achieved by alternately depositing bilayers (BL) of 0.1 wt% polyethylene oxide (PEO) and 0.03 wt% double walled carbon nanotubes (DWNT), dispersed with 0.1 wt% polyacrylic acid (PAA), by the layer-by-layer assembly technique. A 25 BL thin film (~500 nm thick), composed of a PEO/DWNT-PAA sequence, displays electrical conductivity of 19.6 S/cm and a Seebeck coefficient of 60 µV/K, which results in a power factor of 7.1 µW/m·K^2^. The resultant nanocomposite exhibits a crack-free surface up to 30% strain and retains its thermoelectric performance, decreasing only 10% relative to the unstretched one. Even after 1000 cycles of bending and twisting, the thermoelectric behavior of this nanocomposite is stable. The synergistic combination of the elastomeric mechanical properties (originated from PEO/PAA systems) and thermoelectric behaviors (resulting from a three-dimensional conjugated network of DWNT) opens up the possibility of achieving various applications such as wearable electronics and sensors that require high mechanical compliance.

## 1. Introduction

The demand for flexible, stretchable, and wearable materials has been rapidly ever increasing since new technology fields require next-generation electronic devices to be capable of bending and stretching under mechanical deformation. The rapidly developing field of energy harvesting has been gaining immense attention in wearable technologies that are highly desired for smart clothing, flexible sensors’ encapsulation, and electronic textiles [1,2,3,4]. Thus, stretchable and light weight thermoelectric (TE) materials have become important because of their ability to harvest energy from a temperature gradient without moving parts and the need for maintenance [5,6]. The efficiency of a TE material is defined by the dimensionless figure of merit, *ZT* = *S*^2^*σT/к*. Here, *S* is the Seebeck coefficient; σ is the electrical conductivity; *T* is the temperature; *к* is the thermal conductivity. High efficiency energy harvesting thus requires large *S* and high σ, while *к* is low [7]. As an alternative way, the power factor is used to evaluate the TE performance, which is defined as *PF* = *S*^2^*σ*.

The most widely used TE materials are mainly inorganic semiconductors, such as lead and bismuth telluride [8,9,10]. Despite these significant improvements, the widespread usage of inorganic materials is currently prevented by their intrinsic problems including brittleness, scarcity, limited processability, toxicity, and non-flexibility [11,12,13]. On the other hand, conducting polymers and their composites, compounded with carbon nanofillers, have been identified as the most promising alternative materials for the next TE systems [14,15,16]. They possess more desirable properties such as unique mechanical flexibility, low cost, abundance of materials, and typically low thermal conductivity compared to inorganic semiconductors [9,17]. Although the carbon materials, including graphene and carbon nanotubes, exhibit high thermal conductivity (up to 5000 W/m∙K), the carbon nanofiller loaded polymer composites have shown a reasonably low thermal conductivity, ranging from 0.3 to 30 W/m∙K [18,19,20,21].

Several deposition techniques including self-assembled Langmuir–Blodgett, chemical vapor deposition, and other methods have been studied to create ultrathin film devices [22,23,24]. Among these thin film fabrication techniques, the layer-by-layer (LbL) deposition method has attracted widespread attention because it provides precise control over the composition and topography of the final material with nanoscopic order and surface tunable properties [25,26]. Layer-by-layer (LbL) assembly is a simple, but powerful technique to fabricate multifunctional thin films via cyclical adsorption of oppositely charged species. The prominent advantages of the LbL method include simplicity, versatility, and precise controllability for the creation of multilayer thin films [27]. Furthermore, by varying processing conditions (such as ionic strength, pH, types of polyelectrolytes, molecular weight, and temperature) during polyelectrolyte deposition, the physicochemical properties and morphology of multilayers can be simply controlled [28,29,30]. Functionalized multilayers can be created with a variety of combinations of materials such as quantum dots, biological molecules, dendrimers, and carbon nanomaterials [31,32,33,34]. LbL assembly is mainly a result of electrostatic interaction in most cases, but other molecular interactions between the LbL materials, including hydrogen bonds, coordination bonds, charge transfer, hydrophobic interactions, and the combined interaction of the above forces have been shown to be driving forces to build up multilayer films [35,36,37]. LbL films have been engineered in a diverse range of applications, such as drug delivery, sensing, self-cleaning, super hydrophobic surfaces, separation membranes, and energy storage [38,39,40,41].

In spite of significant progress in achieving high performance TE materials, realizing reliable, durable, and stretchable power sources under cyclic mechanical deformation still poses a great challenge. To date, only a few works on elastomeric organic thermoelectric materials have been found in the literature [42,43,44,45]. Finding a new deformable energy source addresses the demands of a variety of emerging applications, including stretchable displays, biointegrated devices for monitoring human health, and many others [46]. Toward addressing a lack of high stretchiness with reasonable TE properties, a new type of multifunctional LbL assembly is demonstrated. In an effort to create stretchable thermoelectric films, we report a simple and environmentally friendly preparation of PEO/double walled carbon nanotube (DWNT)-PAA nanocomposites where PEO and DWNT, dispersed by PAA in water, are alternately deposited. To the best of our knowledge, no study to date has investigated an elastomeric, organic TE nanocomposite using the water based LbL process. The exploration of a stretchable and wearable TE material that utilizes ubiquitous wasted heat as the source of energy can become a practical solution for self-powered electronic devices.

A 25 PEO/DWNT-PAA bilayer (~500 nm in thickness) exhibited an electrical conductivity of 19.6 S/cm and a Seebeck coefficient of 60 µV/K, leading to a power factor of 7.1 µW/m·K^2^. This nanocomposite displayed crack-free surface up to 30% stretching and maintained its TE performance, decreasing only 10% relative to the unstretched one. Furthermore, the electrical conductivity and Seebeck coefficient of this nanocomposite stayed nearly constant after 1000 cycles of bending and twisting. The synergistic combination of each component in the multilayers resulted in compliant and stretchable TE materials. The use of hydrogen bonded PEO/PAA layers rendered the film’s mechanical compliance due to the high elastomeric behavior. Uniformly layered thin films with an extended conjugated DWNT structure created extensive networks that facilitated electron transport. These features rendered the multilayer thin films unique and suitable for wearable electronic devices that could harvest energy from the human body.

## 2. Materials and Methods

### 2.1. Materials

Branched polyethylenimine (BPEI) (Mw = 25,000 g/mol) and polyacrylic acid (PAA) (Mw = 100,000 g/mol) were provided by Sigma-Aldrich (Milwaukee, WI, USA). Polyethylene oxide (PEO) with a molecular weight of 4,000,000 g/mol was purchased from Polysciences (Warrington, PA, USA). Double walled carbon nanotubes (DWNT, XB type: average 1 µm length and 2 nm diameter) were purchased from Continental Carbon Nanotechnologies (Houston, TX, USA). DWNT solutions were prepared by mixing a PAA solution and DWNT in distilled (DI) water at a concentration of 0.1 wt% PAA and 0.03 wt% DWNT. Then, DWNT solutions were dispersed under bath sonication for 20 min, followed by 30 min of tip sonication (Bandelin Sonopuls, Germany), and repeated twice to ensure the suspensions were completely homogenized. The DWNT-PAA solutions were then transferred into a glass centrifuge tube and centrifuged at 4000 rpm for 20 min at room temperature. The supernatant suspension was carefully decanted into a clean glass vial to obtain completely homogenized DWNT. All chemicals were used as received without any further purification unless otherwise stated.

### 2.2. Substrates

Poly(ethylene terephthalate) (PET), with a thickness of 188 µm, was purchased from FilmBank (Gyeonggi-do, Korea). Prior to deposition, the PET films were cleaned with DI water, methanol, and DI water and then dried with compressed air. The cleaned PET substrates were then corona treated using a BD-20C Corona Treater (Electro-Technic Products Inc., Chicago, IL, USA) to impart a negative substrate surface charge, which helped lay down the first primer layer, BPEI [47]. Single side polished silicon wafers (p-type, 100, University Wafer, Boston, MA, USA) were used for characterizing film thickness and surface structure. Silicon wafers were cleaned with DI water, acetone, and DI water and then finally dried with compressed air. Polyurethane (PU) rubber (0.7 mm thick, WooJinPackage, Seoul, Korea) for SEM imaging and thermoelectric behaviors was rinsed with methanol and DI water before being dried with compressed air. Every PU sheet was then treated with a plasma cleaner (Harrick Plasma, PDC 32G-2, Ithaca, NY, USA) at 25 W for 5 min to enhance the adhesion of the first BPEI layer to the substrate.

### 2.3. Multilayer Formation

In an effort to enhance the adhesion between the substrate and multilayers, a single layer of ionically bonded BPEI/PAA was coated before building up PEO/DWNT-PAA thin films. For this primer layer to lay down, the substrates were dipped into 0.1 wt% BPEI and 0.2 wt% PAA solutions for 5 min along with DI water rinse in between each deposition. In order to create PEO/DWNT-PAA bilayer (BL) films, each substrate was first submerged in the non-ionic polymer, PEO, for 5 min and rinsed in a series of three DI water baths for 1 min before the next layer deposition. The DWNT-PAA was then deposited onto the PEO coated substrates by adsorption for 5 min, followed by three rinse baths. After this initial BL was deposited, all immersion times were 1 min for subsequent cycles and repeated until the desired number of BL was achieved.

### 2.4. Characterization of Thin Films

The thickness of BL films deposited on silicon wafers was measured by using a NanoMap-PS contact mode stylus Profilometer. A quartz crystal microbalance (QCM200, Stanford Research Systems, Inc., Sunnyvale, CA, USA) was used to measure the mass of the thin films during multilayer deposition. The multilayer structure was visualized with AFM (Nanostation II^TM^ Surface Imaging Systems, Herzogenrath, Germany) using non-contact mode at a scan rate of 0.5 Hz under ambient conditions. The surface morphology of thin films under various strains was imaged by using an S-4800 Field Emission Scanning Electron Microscope (FE-SEM) (Hitachi, Japan). The elastic modulus was obtained using nano-indentation mode (Nanostation II^TM^ AFM, Herzogenrath, Germany) and calculated using force curve analysis in conjunction with the Hertz model.

### 2.5. Thermoelectric Measurements

The electrical resistance of the multilayers at 0% strain was measured with a 4 point probe (CMT-100S, Advanced Instrument Technology, Suwon, South of Korea) having a 0.4 mm probe tip diameter and a 0.72 mm tip spacing. The Seebeck coefficient measurement was determined by using a home built 4 point probe setup where two copper wires and two T-type thermocouples were used to measure electrical voltage and temperature difference, respectively. Prior to measurements, a silver paste was applied to the samples to avoid electrical contact resistance. The thermoelectric voltage across the films under 8 temperature differentials between −10 and 10 K was recorded by LabVIEW. The reported Seebeck coefficient was obtained from the linear slope of the ∆T vs. ∆V relation. The electrical conductivity and Seebeck coefficient values reported represented an average of 5 measurements on 3 independent samples. Resistance of the thin films under 0–50% strains was measured using a two point multimeter with 1 mm tip spacing. The multilayers (~500 nm thick) were stretched up to 50% with the strain rate of 0.42 cm/s, and the Seebeck coefficient was measured while each sample was held.

## 3. Results

**Figure 1:**
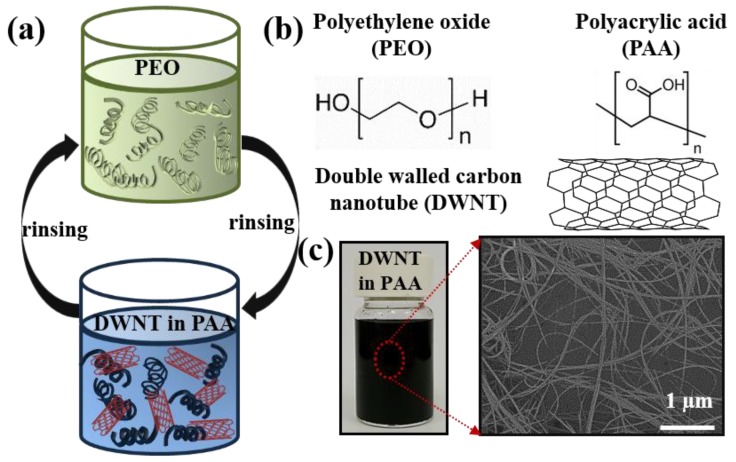
(**a**) Schematic of the layer-by-layer process for PEO/DWNT-PAA thin films. (**b**) Molecular structures of PEO, PAA, and DWNT. (**c**) Optical image of aqueous of DWNT stabilized by PAA in water. A top view image in SEM of the corresponding suspension cast onto silicon wafer is shown next to DWNT suspensions in PAA.

Figure 1a shows a schematic of the layer-by-layer (LbL) deposition process of the multi-dimensional nanocomposite film consisting of polymers and DWNT from aqueous solutions. Figure 1b depicts the chemical structures of each component used in this work, one being the polymer pairs, which were studied as a stretchable system, and the other being the conductive nanofiller, DWNT. The multilayer assembly was done by alternately depositing PEO (as a hydrogen bond acceptor) and DWNT, stabilized in PAA (as a hydrogen bond donor) [48,49]. Unlike PEO being unaffected by the solution pH, high sensitivity to the assembling pH of PAA chains created a favorable pH window where PEO/PAA systems were systematically growing. For example, the repulsive force exerted between carboxylate groups at pH > 3.5 or self-association due to complete protonation of carboxylic acid groups at pH < 2 on PAA chains frustrated the PEO/PAA assembly [50]. In this work, each solution was deposited at a pH of 2.8 in which some protonated carboxylic acid groups in the partially charged PAA chains were available for hydrogen bonding with the ether oxygen in PEO [51,52]. DWNT nanoparticles were enveloped by the PAA and PEO [53]. Sonicating DWNT in a negatively charged PAA solution resulted in a homogenous black suspension, as shown in Figure 1c. An optical image of the aqueous DWNT-PAA showed a stable colloidal dispersion at room temperature with little or no precipitation observed for an extended period of time. A scanning electron microscope (SEM) image confirmed the well dispersed carbon nanotubes in the PAA solutions.

### 3.1. Growth Behavior

**Figure 2:**
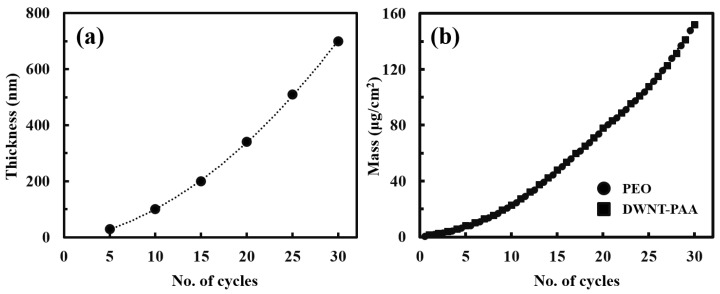
(**a**) Thickness and (**b**) mass of PEO/DWNT-PAA bilayer systems as a function of cycles of deposition.

Figure 2a shows the thickness as a function of PEO/PAA bilayers (BL). The thickness increased with an increase in the number of BL, exhibiting an exponential growth behavior. The thickness profile was dictated by the employment of PAA in each deposition cycle, which induced “in-and-out” diffusion of PEO and PAA during assembly. As is well documented, PEO/PAA films assembled at a pH between 2 and 3 grow exponentially during which an immense amount of diffusive intermixing takes place during deposition instead of staying at the position where they were initially deposited [52,54]. For the remainder of this paper PEO/DWNT-PAA (0.1 wt%) refers to a sample constructed from DWNT dispersed in 0.1 wt% PAA, as distinguished from PEO/DWNT-PAA (0.2 wt%) in which DWNT is stabilized in 0.2 wt% PAA. PEO/DWNT-PAA (0.1 wt%) films exhibited a lower growth rate relative to 0.2 wt% PAA based counterparts. For instance, the present system attained a thickness of 700 nm at 30 BL, while PEO/DWNT-PAA (0.2 wt%) had 950 nm at the same layers (Figure S1a). This indicated that controlling the concentrations of polyelectrolytes could induce significant changes in the thickness, as previously studied with other cases such as the pH, temperature, ionic strength, and molecular weight of the LbL thin films [55,56,57]. Figure 2b shows the mass of PEO/DWNT-PAA films. Likewise, the mass increased exponentially as the number of BL deposited increased. This further confirmed that each component was deposited with constant composition during growth. Based on the film thickness (from the profilometer) and mass (from QCM), the density of the PEO/DWNT-PAA BL film was calculated to be ~2.17 g/cm^3^.

### 3.2. Thermoelectric Properties

**Figure 3:**
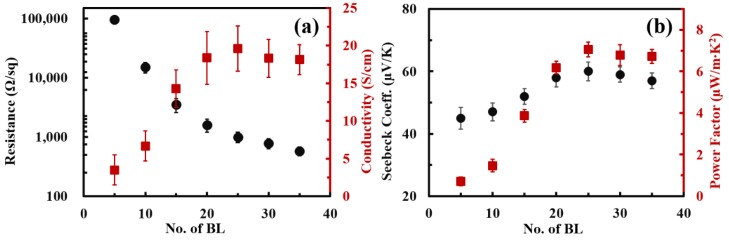
(**a**) Sheet resistance and electrical conductivity and (**b**) Seebeck coefficient and power factor of PEO/DWNT-PAA as a function of bilayers (BL) deposited.

The electrical resistance and conductivity of PEO/DWNT-PAA films were measured with a four point probe system. The sheet resistance of PEO/DWNT-PAA BL films decreased with increasing the number of layers deposited. (Figure 3a). This is simply explained by the fact that the amount of DWNT coated on the PET increased as the number of BL increased, as confirmed by the result of the thickness and mass (Figure 2). The sheet resistance was as large as 95 KΩ/sq at 10 BL, but it significantly decreased with the number of deposited layers to achieve 580 Ω/sq at 25 BL (~500 nm in thickness). The electrical conductivity of this system was obtained by taking the reciprocal of electrical resistivity in which the sheet resistance and film thickness of the material was multiplied. When the layers were added, the electrical conductivity dramatically increased from 3.5 S/cm at five BL to 19.6 S/cm at 25 BL. The gradual increase of the electrical conductivity from 3.5 to 19.6 S/cm in PEO/DWNT-PAA films revealed that the density of the intersecting pathways of electrons increased in proportion to the deposition cycles [16]. Further increasing of the layers then led to a leveling off in conductivity, indicating that the electrical conductivity was largely affected by the film structure rather than the amount of conducting materials deposited [58,59].

By lowering the concentration of PAA from 0.2 to 0.1 wt%, the electrical conductivity of the PEO/DWNT-PAA (0.1 wt%) composites was nearly 30 times as large as that of the recently reported system (PEO/DWNT-PAA (0.2 wt%)) (Figure S2) [53]. The exact mechanism is not clear at this time, but we believe that the electrical conductivity presumably reflected how each component was incorporated into the conductive structure rather than how much conducting material was deposited [58]. The film density of 0.2 wt% PAA based multilayers was calculated to be 2.55 g/cm^3^ (Figure S1). This means that the deposition rate of DWNT was much greater in the previous system relative to the present one. The greater rate of DWNT deposition in the 0.2 wt% PAA based system resulted in randomly dispersed, stacked DWNT within the multilayers, which was not favorable for efficient electrical transport (Figure S3). However, the current system was expected to have a more ordered and well networked structure of nanotubes, which significantly enhanced the electrical conductivity, as compared to the previous one (0.2 wt% PAA based system).

Figure 3b shows the Seebeck coefficient and power factor of these multilayer thin films as a function of the number of layers deposited. The positive value of the Seebeck coefficient proved that the films were p-type with hole dominated carrier transport. The Seebeck coefficient was 45 μV/K at five BL and increased steadily as the layers were added, attaining a maximum value of 60 μV/K at 25 BL. After reaching a peak, the value then settled to around 58 μV/K. It was interesting to see that both the electrical conductivity and Seebeck coefficient increased simultaneously with increasing layers, which was different from the conventional inorganic TE materials that displayed an inverse relationship. The decoupled behavior in the TE properties could be attributed to improved carrier mobility as previously studied in the multilayer systems [60,61]. Based on the measured electrical conductivity and Seebeck coefficient, the power factor (*PF* = *S*^2^∙*σ*) was calculated as a function of layers deposited. The *PF* in the PEO/DWNT-PAA films exhibited an increase with adding layers, in a similar manner to electrical conductivity. This indicated that the large increase in the conductivity was responsible for the power factor in this material. At 25 BL (~500 nm in thickness), this film had a power factor of 7.1 μW/m∙K^2^. Inaccurate characterization of the thermal conductivity of the thin films (<1 μm) made it difficult to measure the total in-plane thermal conductivity in this work. We believe that the thermal conductivity would range from 0.4 to 24.6 W/m∙K based on other reports [62,63,64]. However, lower thermal conductivity was expected because of the phonon scattering through numerous interfaces created from the nanostructured layers [65].

### 3.3. Multilayer Structure

**Figure 4:**
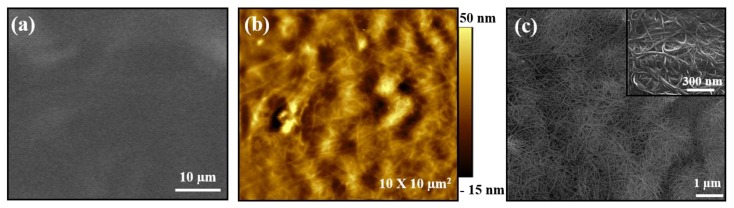
(**a**) SEM image of two bilayer PEO/PAA and (**b**) AFM and (**c**) SEM images of two bilayer PEO/DWNT-PAA assemblies. The inset in the SEM image of two PEO/DWNT-PAA BL films shows the higher resolution of DWNT.

In order to better understand the thermoelectric properties of PEO/DWNT-PAA nanocomposites, the microstructure of the multilayers was visualized using AFM and SEM, as shown in Figure 4. While the PEO/PAA BL system displayed smooth and featureless structure (Figure 4a), the AFM height image of a 2 BL film exhibited uniformly dispersed DWNT in the PEO/DWNT-PAA films (Figure 4b). An image of SEM of PEO/DWNT-PAA composites in Figure 4c shows single nanotubes and their bundles interwoven with one another. A 3D architecture with polymer-like entanglements of nanotubes was created with only a few deposition cycles. This well dispersed nanotube structure gave rise to the formation of an excellent conductive network. The inset image further confirmed evidence about highly conjugated π-network with excellent coverage.

### 3.4. Influence of Strain on Thermoelectric Performance

**Figure 5:**
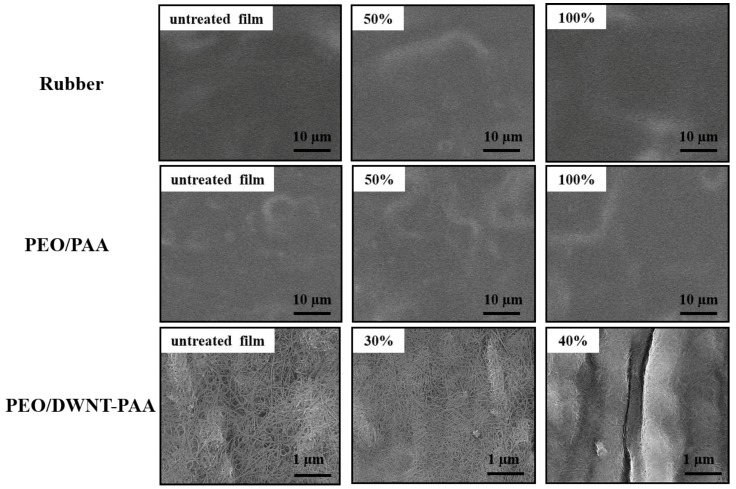
SEM surface images of uncoated PU rubber and 25 bilayer PEO/PAA and PEO/DWNT-PAA assemblies, on 0.7 mm thick polyurethane rubber, before and after being subjected to varying levels of strain.

To investigate the strain stability of the nanocomposite film, the changes in the morphology during the stretchiness were evaluated using the SEM images, as shown in Figure 5. No cracks were observed on the uncoated polyurethane (PU) substrate when stretched to 100%. The 25 PEO/PAA BL film (~500 nm thick) had a featureless surface. By stretching the PEO/PAA thin films up to 100% strain, there were no cracks on the surface. In the case of 25 BL PEO/DWNT-PAA films, the multilayers had a crack-free surface with uniformly dispersed nanotubes at 0% strain. This film was entirely free of cracks under a strain of 30%, but crack-like defects formed above this level. Interfacial strain localization was responsible for these defects, which was observed in PEO/PAA systems [66].

**Figure 6:**
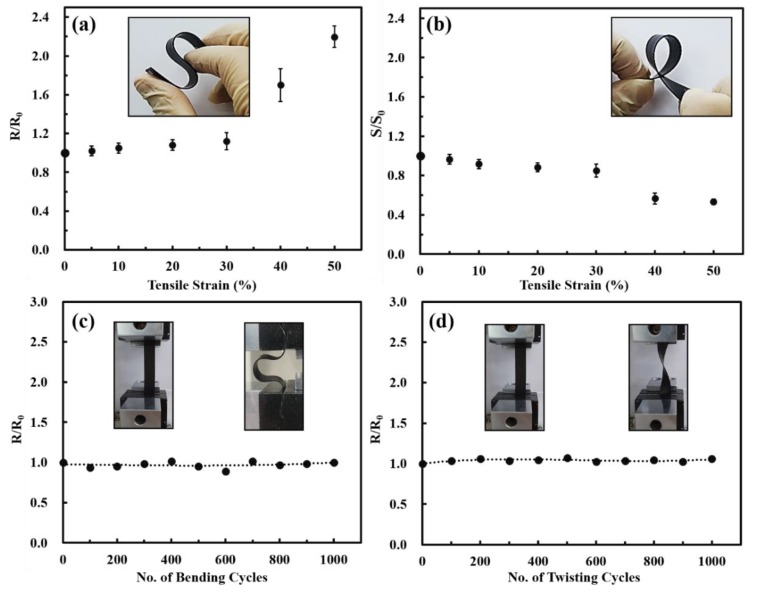
(**a**) Normalized resistance and (**b**) Seebeck coefficient of ~500 nm thick 25 bilayer PEO/DWNT-PAA as a function of tensile strain. Normalized resistance of 25 bilayers of PEO/DWNT-PAA in cyclic (**c**) bending and (**d**) twisting tests. Each inset photo shows the bending and twisting process. The normalized Seebeck coefficient of 25 bilayers of PEO/DWNT-PAA by cyclic bending and twisting is shown in the Supporting Information (Figure S4).

The electrical conductivity and Seebeck coefficient of the PEO/DWNT-PAA BL films were measured during the stretching of the nanocomposites, and the results are shown in Figure 6a,b. The initial resistance and Seebeck coefficient at unstretched state are referred to as R_0_ and S_0_, respectively. Figure 6a shows the normalized resistance (R/R_0_) of the multilayers against applied tensile strain. The nanocomposites moderately responded to the applied strain of 30% with a slight increase in resistance, but increased dramatically above this level. The S/S_0_ as a stretching cycle indicated that the 30% stretched films exhibited a gradual decrease in the Seebeck coefficient, with a greater rate of decrease for the larger strain (Figure 6b). The decrease in thermoelectric properties beyond 40% strain was presumably due to the cracks/defects caused by contact loss in the conjugated nanotube network. We believe that this is the first report on a stretchable organic TE nanocomposite prepared with LbL assembly. Each inset photo in Figure 6a,b clearly confirmed that the PEO/DWNT-PAA multilayers revealed excellent flexibility.

To verify the long term stability of the multilayers, simple cycling bending and twisting tests were performed. The sheet resistance and Seebeck coefficient of the 25 BL PEO/DWNT-PAA films showed little sensitivity to bending (Figure 6c and Figure S4a). The resistance was almost unchanged after cyclic bending 1000 times, and only a tiny decrease was observed. This means that the DWNT conjugated network could effectively retain its original structure after bending deformation. Both electrical resistance and Seebeck coefficient showed little degradation upon twisting (Figure 6d and Figure S4b). The resistance increased only 10% and the Seebeck coefficient decreased around 15% during 1000 cycles of twisting, indicating excellent mechanical properties, as well as high electrical stability. These results indicated that the 3D TE materials with highly elastic conductors could be useful in a stretchable and wearable power supply.

## 4. Discussion

**Figure 7:**
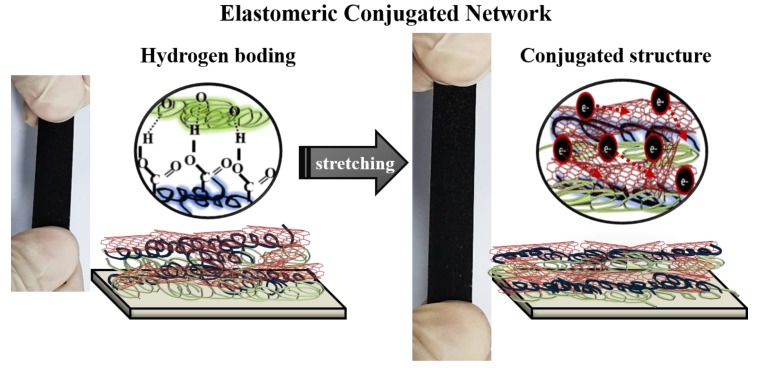
Schematic of the elastomeric conjugated network in PEO/DWNT-PAA nanocomposite thin films.

The high performance in conductive elastomers with TE properties originates from the synergism through fully utilizing the advantages of nanoscale engineering via the LbL approach, without sacrificing their individual traits (Figure 7). The high mechanical compliance of the PEO/DWNT-PAA multilayers was mainly due to the flexible elastomeric behavior in PEO/PAA layers that accommodated large bendability, twistability, and stretchability. It is known that PEO/PAA LbL films behave as flexible elastomeric blends because of the weak bond strength and high chain mobility between PEO and PAA layers [67]. In striking contrast to ionically bonded LbL systems (e.g., BPEI/PAA or BPEI/montmorillonite) that suffer from cracks upon strains (<5%) because of their brittle nature (E ~ 200 GPa), hydrogen bonded ductile PEO/PAA systems exhibit no cracks or defects even at 100% strain because of their rubbery nature at room temperature (E ~ 100 MPa) [52,68,69]. The incorporation of carbon nanofillers, DWNT, into PEO/PAA layers added rigidity to the multilayers, which in turn degraded the stretchability and cracks above 40% strain. The elastic modulus (400 MPa) of PEO/DNWT-PAA was shown to be much higher than that of PEO/PAA (~30 MPa) (Figure S5).

The three-dimensional (3D) carbon network originated from DWNT in these multilayer thin films imparted a high electrical conductivity of 19.6 S/cm and a Seebeck coefficient of 60 μV/K, which resulted in a power factor of 7.1 μW/m∙K^2^. The relatively high electrical performance of the multilayers could be explained by the fact that the π-π interaction between the DWNT network would give rise to orderly, packed, conjugated structures, which provided an efficient pathway for carrier transport [16,59,70]. The combined formation of elastomeric blends (PEO/PAA layers) and extended conjugated DWNT networks resulted in high electrical properties at high strain levels of 30% (Figure 6). The elastomeric matrix, PEO/PAA layers, could help distribute the stress more uniformly, helping electric contacts between the carbon nanofillers [67,71]. When stretched, the stress imposed by external strains could be mitigated through bond slip and reorientation along the polymers’ and carbon nanofillers’ interfaces [72]. In other words, upon exposure to the strains, the carbon nanotubes network in PEO/PAA layers could slide past one another without losing contact and maintain effective conductive junctions. Large strain deformation (>40%) caused the weakening and detachment of the contacts between carbon nanofillers, triggering conductive pathway disruption and consequently leading to a large increase of resistance and a decreased Seebeck coefficient (Figure 6).

## 5. Conclusions

This work demonstrated a strategy to develop stretchable thermoelectric thin films via layer-by-layer (LbL) assembly. We stressed the important finding about a synergistic self-assembly of organic nanocomposites that possess high stretchability (originated from an elastomeric PEO/PAA layers) and thermoelectric properties (generated from a 3D conjugated DWNT network). A 25 BL film (~500 nm thick), consisting of PEO, DWNT, and PAA, achieved an electrical conductivity of 19.6 S/cm and a Seebeck coefficient of 60 µV/K, which translated to a power factor of 7.1 μW/m∙K^2^ at room temperature. The multilayer thin films were stretched up to 30% without any cracks or defects observed on the surface and intact thermoelectric behaviors. This film showed a high stretchability (~30% strain) and stability (no discernible changes in structure and thermoelectric performances under 1000 bending and twisting cycles). The ability to impart stretchiness with thermoelectric performance to thin films fabricated via LbL deposition can foresee possible application for a variety of body worn electronics that require bendability, stretchability, and wearability.

## Supplementary Materials

The following are available online at https://www.mdpi.com/2079-4991/10/1/41/s1: Figure S1: (**a**) Thickness and (**b**) mass of PEO/DWNT-PAA bilayer systems with different concentrations of PAA (0.1 and 0.2 wt%) as a function of deposition cycles. Figure S2: Electrical conductivity of PEO/DWNT-PAA assembled with 0.1 (filled square) and 0.2 (open square) wt% as a function of bilayers deposited. Figure S3: Schematics of carrier transport across the nanotubes in the PEO/DWNT-PAA nanocomposites where DWNT is dispersed with 0.1 and 0.2 wt% PAA, respectively. Figure S4: Normalized Seebeck coefficient of ~500 nm thick 25 BL of PEO/DWNT-PAA in cyclic (**a**) bending and (**b**) twisting tests. Figure S5: Elastic modulus of 25 PEO/PAA BL and PEO/DWNT-PAA BL films deposited on a Si-wafer.
