# Supplementary material for: Organic Thermoelectric Multilayers with High Stretchiness
Source: Nanomaterials (Basel). 2019 Dec 23;10(1):41. doi: 10.3390/nano10010041 (PMC7023331; doi:10.3390/nano10010041)
Supplement: Supplementary file 1 [file nanomaterials-10-00041-s001.pdf]

nanomaterials

MDPI

Supporting Information

Organic Thermoelectric Multilayers with High Stretchiness

Chungyeon Cho [1,*] and Jihun Son [1]

Department of Carbon Convergence Engineering, College of Engineering, Wonkwang University, Iksan 54538, Jeonbuk, Korea; sjhoon14@wku.ac.kr

* Correspondence: cncho37@wku.ac.kr; Tel.: +82-63-850-7274

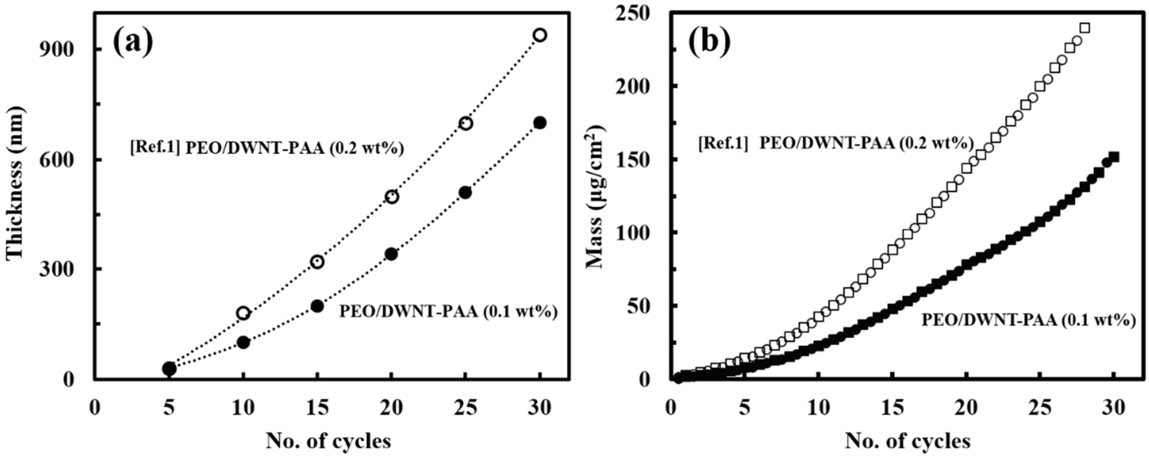

Figure S1. (a) Thickness and (b) mass of PEO/DWNT-PAA bilayer systems with different concentrations of PAA (0.1 and 0.2 wt%) as a function of deposition cycles.

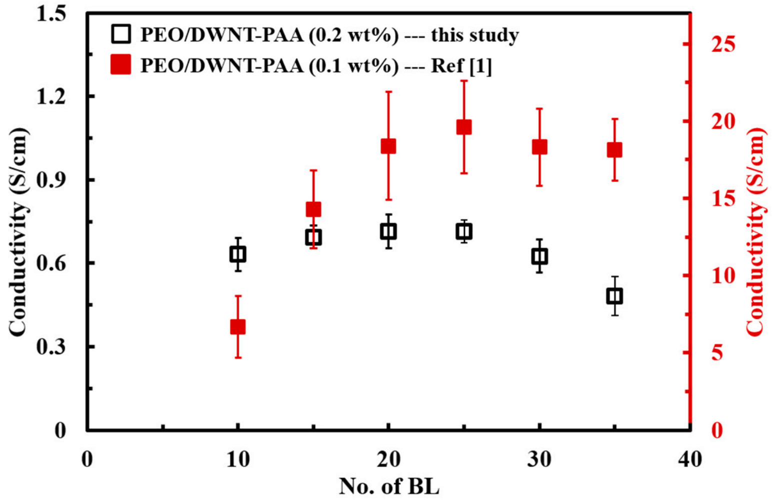

Figure S2. Electrical conductivity of PEO/DWNT-PAA assembled with 0.1 (filled square) and 0.2 (open square) wt% as a function of bilayers deposited.

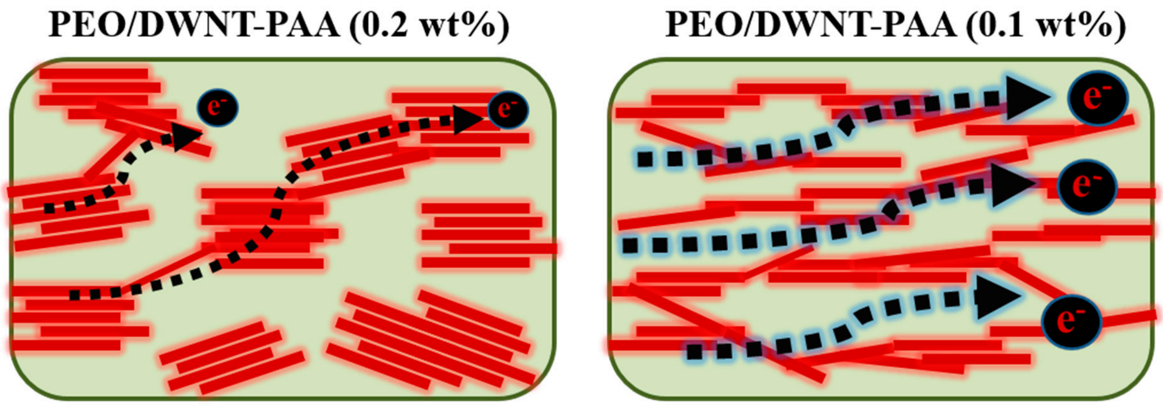

Figure S3. Schematics of carrier transport across the nanotubes in the PEO/DWNT-PAA nanocomposites where DWNT is dispersed with 0.1 and 0.2 wt% PAA, respectively.

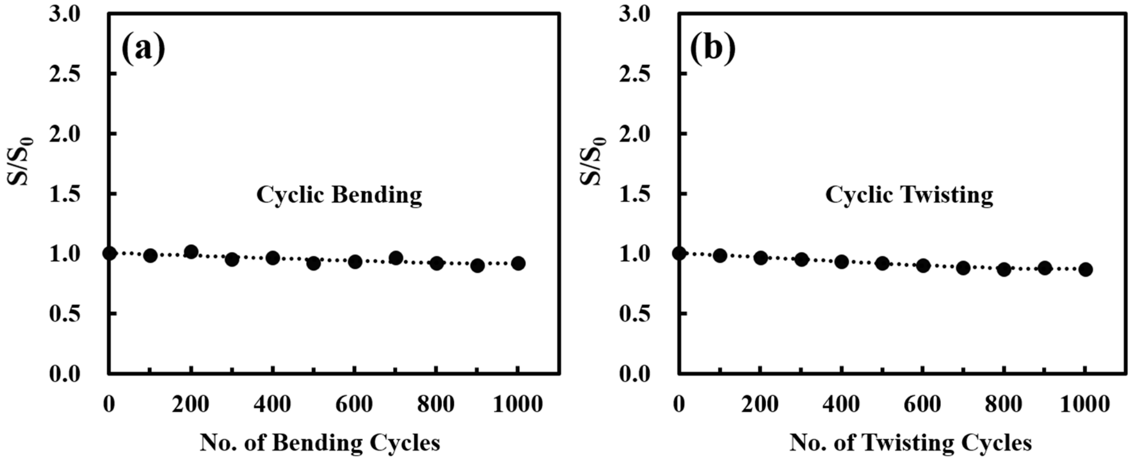

Figure S4. Normalized Seebeck coefficient of ~ 500 nm thick 25 bilayer of PEO/DWNT-PAA in cyclic (a) bending and (b) twisting tests.

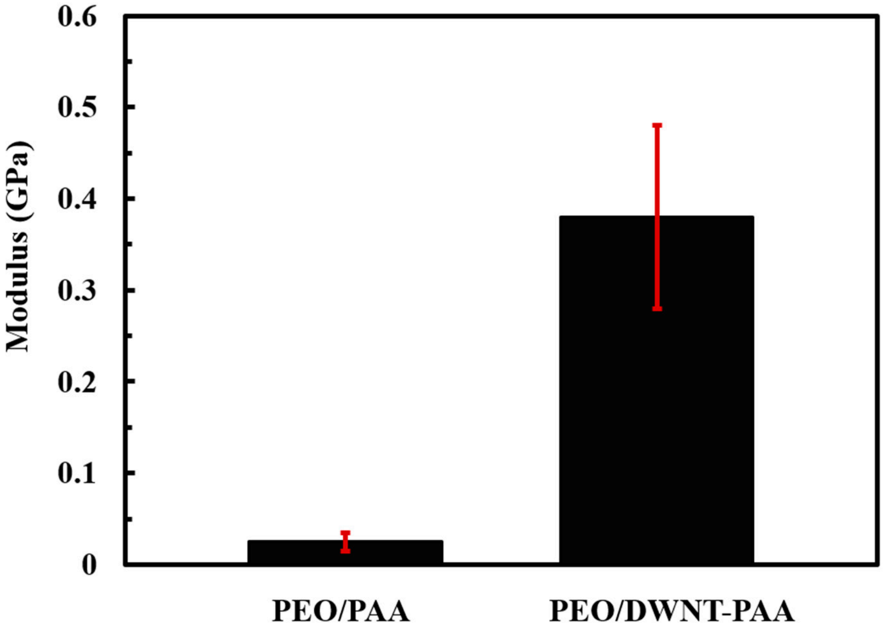

Figure S5. Elastic modulus of 25 bilayer PEO/PAA BL and PEO/DWNT-PAA films deposited on a Si-wafer.

References

1. C. Cho, Y. Song, R. Allen, K.L. Wallace, J.C. Grunlan, Stretchable electrically conductive and high gas barrier nanocomposites, Journal of Materials Chemistry C 6(8) (2018) 2095-2104.
